# A Dual-Stream Cross AGFormer-GPT Network for Traffic Flow Prediction Based on Large-Scale Road Sensor Data

**DOI:** 10.3390/s24123905

**Published:** 2024-06-17

**Authors:** Yu Sun, Yajing Shi, Kaining Jia, Zhiyuan Zhang, Li Qin

**Affiliations:** 1 School of Electronic and Information Engineering, Beijing Jiaotong University, Beijing 100044, China; 21211163@bjtu.edu.cn (Y.S.); 21211356@bjtu.edu.cn (K.J.); 2 Jeme Tienyow Honors College, Beijing Jiaotong University, Beijing 100044, China; 21251131@bjtu.edu.cn; 3 Key Laboratory of Communication and Information Systems, Beijing Jiaotong University, Beijing 100044, China; 4 PLA Academy of Military Science, Beijing 100044, China

**Keywords:** traffic flow prediction, prompt engineering, dual-stream cross, GFormer-GPT

## Abstract

Traffic flow prediction can provide important reference data for managers to maintain traffic order, and can also be based on personal travel plans for optimal route selection. On account of the development of sensors and data collection technology, large-scale road network historical data can be effectively used, but their high non-linearity makes it meaningful to establish effective prediction models. In this regard, this paper proposes a dual-stream cross AGFormer-GPT network with prompt engineering for traffic flow prediction, which integrates traffic occupancy and speed as two prompts into traffic flow in the form of cross-attention, and uniquely mines spatial correlation and temporal correlation information through the dual-stream cross structure, effectively combining the advantages of the adaptive graph neural network and large language model to improve prediction accuracy. The experimental results on two PeMS road network data sets have verified that the model has improved by about 1.2% in traffic prediction accuracy under different road networks.

## 1. Introduction

In the current society, with the high development of the economy and the strengthening of urbanization and population mobility, the complexity and challenge of the traffic system are becoming more and more prominent [1]. Effective and real-time traffic flow prediction is of great significance for alleviating traffic congestion, reducing traffic accidents, optimizing resource allocation, ensuring traffic safety, and formulating reasonable traffic planning policies. The traffic flow prediction algorithm, as the most critical research field in the context of intelligent transportation, has received great attention from scholars [2,3,4].

Recently, many traffic prediction models have been proposed by scholars. The deep neural network has the advantages of high precision, powerful non-linear relationship processing ability, strong generalization ability, ability to handle large-scale data, real-time prediction and response, and strong adaptability in traffic prediction, which makes deep neural networks a research hotspot in the field of traffic prediction [5]. For instance, Ref. [6] used stacked autoencoder (SAE) deep models to mine historical traffic information, which can effectively capture potential spatiotemporal dependencies. Ref. [7] combined LSTM and the attention mechanism from multiple scales to obtain important factors affecting traffic flow, thereby obtaining better prediction results. Ref. [8] proposed a DBN-SVR traffic prediction model, which has smaller errors than conventional DBN prediction models and other commonly used prediction models. As a variant of RNN, LSTMs can prevent long-term information forgetting and are widely proven to be effective predictive models. Ref. [9] used LSTM and CNN to separately capture the spatiotemporal dependence features and effectively integrate the input of traffic speed and flow using attention mechanisms, intuitively demonstrating the solvability of the DNN-BTF model. Ref. [10] divided road segments into different levels using ideal solution similarity ranking preference technology, and then used convolutional LSTM networks to spatiotemporally mine key road segments, which can accurately predict various states of key road segments. Ref. [11] proposed a CNN traffic prediction network capable of spatiotemporal analysis. This network analyzes the spatiotemporal correlation of progressiveness data before using CNN feature mining, which can select spatiotemporal features and improve the effectiveness of prediction algorithms. Ref. [12] proposed a new parallel processing framework combined with a distributed LSTM traffic prediction model, which improves the accuracy, efficiency, and scalability of predictions. Ref. [13] used hierarchical clustering to split traffic flow up into multiple parts, then calculated the spatial correlation through standard Euclidean space. Then, LSTM was used to mine the information of the most relevant sections of the predicted data, which has high prediction accuracy. Ref. [14] used a dual-branch gated convolutional network to extract the spatiotemporal dependence of historical traffic data, and designed an attention mechanism that only increases the width of the model to weight the hidden features, which ensures high prediction accuracy while increasing less computation time. Ref. [15] introduced the attention mechanism into the LSTM for traffic flow prediction. This helps the network model allocate different attention to different weight inputs, pay attention to key and essential information, and increase prediction accuracy. Ref. [16] combined LSTM networks with ensemble learning’s XBoost structure to forecast traffic flow, avoiding overfitting and improving the generalization of prediction models.

Traditional convolutional or recurrent neural network algorithms are only applicable to the feature extraction of Euclidean space data, and are not applicable to the graph data of non-Euclidean space generated by complex traffic networks. The graph neural network [17,18] can realize the information of neighbors of each node to mine their hidden features through various aggregation methods, and can well characterize the spatial topology of the traffic network, so it has a good prospect for the traffic network data mining task in non-Euclidean space. For example, [19] was the first to apply spatiotemporal graph convolution (STGCN) to traffic prediction, which uses graph convolution and gated convolution to mine hidden features with better accuracy compared to ordinary convolutions. Ref. [20], for the first time, integrated attention into the spatiotemporal graph neural network (ASTGCN). It mines the spatiotemporal correlation through three branches of different time attributes, and uses spatiotemporal attention to weight the hidden features of each layer in each branch, which further improves the prediction accuracy. Ref. [21] designed a special time–graph convolutional network (T-GCN) traffic prediction method, which combines the advantages of GCN and GRU to effectively capture the dynamic spatiotemporal correlation of traffic data, and the prediction effect is better than the advanced model proposed before. Ref. [22] designed a graph multiple attention network (GMAN) based on the encoder–decoder structure to predict the road network conditions of different duration. In this model, spatial attention and temporal attention mechanisms were combined by the gating mechanism to weight the spatiotemporal embedding features. Experiments on real data machines showed that it was effective in long-term prediction tasks. Ref. [23] designed a spatiotemporal synchronous graph convolutional network (STSGCN), which for the first time mined spatiotemporal correlation synchronously by establishing a local spatiotemporal graph of traffic, and significantly improved the prediction accuracy compared with the asynchronous mining of spatiotemporal correlation. Ref. [24] proposed a data-driven approach to generating “time graphs” to compensate for correlations that may not be reflected in spatial graphs with good performance for long-term prediction. Ref. [25] used a novel transformer network (STTN) to extract spatiotemporal dependencies of traffic feature, which effectively extends spatiotemporal relationships over long distances. Ref. [26] proposed a method of automatically obtaining the spatiotemporal state and spatiotemporal dependency in data using a multi-graph GAN. This method can obtain real-time traffic prediction results through GAN constraints. Ref. [27] fused the spatiotemporal characteristics of traffic flow through the spatiotemporal multi-graph convolutional network with cross attention, effectively reducing the prediction error. Ref. [28] designed a special spatiotemporal GCN mine global and local spatial relationships and integrated multi-granularity temporal dynamic relationships. In addition, it can fully utilize the semantic information of traffic data and achieve good predictive performance. Ref. [29] proposed a new model for learning the spatiotemporal relationships of traffic data, which can dynamically express temporal- and spatial-related features in a graphical manner, fully tapping into the inherent connections of time and space, and effectively improving accuracy. Ref. [30] designed an adaptive adjacency matrix calculation method combined with GCN to mine the dynamic spatial relationships of road information. Compared to using a fixed adjacency matrix local aggregation road network node hidden feature local method, it has better accuracy and adaptability. Ref. [31] proposed the spatiotemporal graph neural controlled differential equation (STG-NCDE) method for traffic prediction. In the process of converting dynamic graphs into time series, each point is converted into a sequence, and then ODE processing is performed on each sequence to effectively improve the accuracy. Ref. [32] used multiple transformer encoders to progressively predict future traffic conditions and adaptively select the optimal model. Ref. [33] enhanced DetectorNet with transformer, and excavated long and short time correlations and dynamic spatial correlation through multi-perspective spatiotemporal attention, and made accurate predictions. Ref. [34] used a temporal transformer network to mine the correlation between the recent and cycle time of traffic flow and generated the final predicted value in combination with a spatial transformer. Ref. [35] proposed a new model named Trafformer for traffic prediction, which unifies spatial and temporal information in one transformer-style model, which enables it to catch complex spatial–temporal dependencies. Ref. [36] fully considered the spatiotemporal heterogeneity when performing traffic prediction tasks, and used a causal spatiotemporal synchronous graph convolutional network to mine spatiotemporal correlations, achieving the best prediction results. Ref. [37] generated adjacency matrices through traffic flow matrices, and then combined attention mechanisms and graph convolutional networks to build a transformer encoder as a hidden feature extractor to mine spatiotemporal correlations, making the prediction model more effective.

Although these existing prediction models can be applied to irregular traffic network data, they still have shortcomings in extracting and utilizing the depth features of historical data, which mainly contain two parts: (1) In the feature extraction process, the influence of the interaction information between various observable traffic parameters on the prediction accuracy is ignored. (2) Traffic information spreads along two dimensions of space–time and space. Utilizing the effective spatiotemporal dependencies of historical traffic data remains a challenge. Regarding the above shortcomings, we designed a dual-stream cross AGFormer-GPT network with prompt engineering for traffic flow prediction. The uniqueness of this article lies in the following.

Firstly, our model designs a reminder engineering module that embeds historical traffic speeds and occupancy rates into a reminder sequence using cross attention. Then, the reminder sequence is effectively integrated into the historical traffic flow sequence, enabling more useful historical information to be utilized in subsequent spatiotemporal correlation mining and improving the prediction accuracy of the prediction model.

Secondly, our model introduces a dual stream crossover network that can simultaneously learn spatial and temporal information to improve performance. This dual flow method can avoid the sequential problem of extracting spatial and temporal correlations, and increase the non-linear fitting ability to better capture the complexity of traffic flow. It uses a fine-tuning GPT2 model to mine temporal correlations and an adaptive graph transformer network to mine spatial correlations, providing a thorough understanding of spatial and temporal dynamics.

Finally, our model is compared with several advanced prediction methods on two real data sets of PeMS. The experimental results show that the proposed method has better performance than others.

## 2. Preliminary

This section describes the symbolic representation of variables and the definition of related concepts in detail.

**Spatial topology** The spatial topology of the traffic network is a graph structure G=(V,E), where $V\in R^{N}$ is the set of nodes, each representing the data sensor’s spatial position. *N* is the number of data sensors. *E* is the set of edges, and $A\in R^{N\times N}$ is the adjacency matrix of the graph structure *G*. **Feature matrix.** There are three kinds of observable traffic network information as the features of the nodes: traffic flow $X^{f}\in R^{N\times T}$, traffic occupancy $X^{o}\in R^{N\times T}$, and traffic speed $X^{s}\in R^{N\times T}$. $X_{t}^{f}$, $X_{t}^{o}$, and $X_{t}^{s}$ represent the feature matrix composed of three information features of network nodes at time *t*.

Among them, traffic flow and traffic occupancy data can be obtained in real time by sensors installed on the road (such as a ring detector, video detector, etc.), providing real-time data support for the traffic management system. Traffic speed data can be obtained by sensors such as floating vehicle technology, GPS positioning technology, and microwave radar tachometer to provide real-time and accurate traffic speed information for traffic management departments.

The traffic flow prediction problem defined in this paper is described as follows. Learning a non-linear function F (prediction model) calculating the traffic flow series for the next period,

(1) $$\left[X_{t+1}^{f},\cdots ,X_{t+\tau_{1}}^{f}\right]=F\left\{\begin{array}{c} {}[X_{t-\tau_{2}+1}^{f},\cdots ,X_{t}^{f}],\\ \left[X_{t-\tau_{2}+1}^{o},\cdots ,X_{t}^{o}\right],\\ \left[X_{t-\tau_{2}+1}^{s},\cdots ,X_{t}^{s}\right],\\ G \end{array}\right\}$$

where $\tau_{1}$ is the prediction horizon and $\tau_{2}$ is the history length of the input matrix.

## 3. Methodology

**Figure 1:**
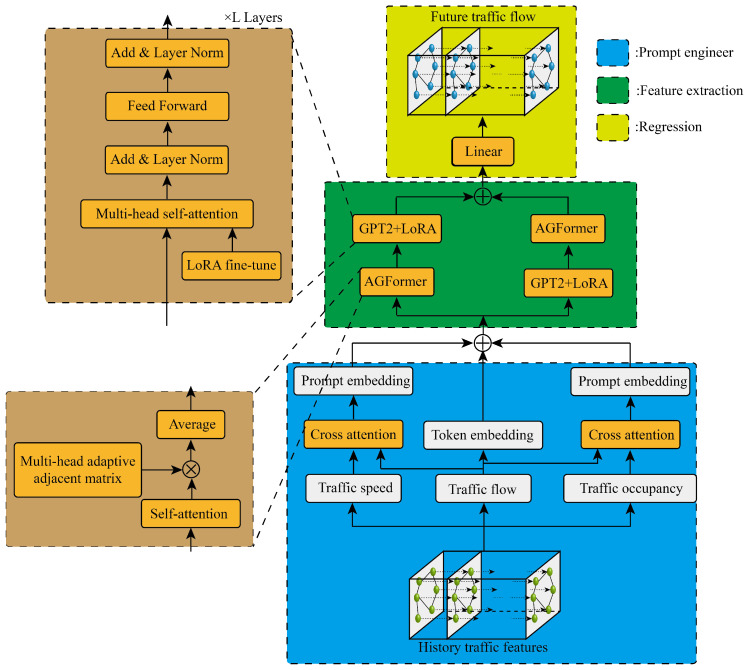
The structure of the prediction model.

Figure 1 shows the specific structure of the dual-stream cross AGFormer-GPT network with prompt engineering proposed in this paper, which mainly includes three parts: prompt engineering, feature extraction and regression. The prompt engineering part can integrate historical traffic flow with other observable related parameters, the feature extraction part synchronously excavates spatiotemporal correlation through a dual-stream cross structure, and the regression part obtains the final predicted value through a linear layer. Next, we will provide a detailed description of the specific contents of the three parts.

### 3.1. Prompt Engineer

**Figure 2:**
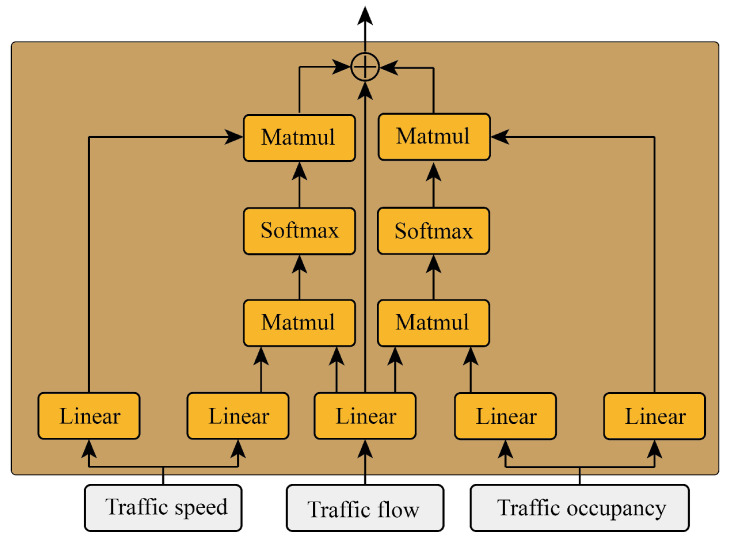
The structure of the prompt engineer.

In traffic theory, traffic speed and traffic occupancy have a key impact on the change in traffic flow, and these two parameters are easy to observe. The suggested project proposed in this paper is to integrate the effective information of the two parameters of traffic speed and occupancy into traffic flow information through two cross-attention mechanisms, and the module is shown in Figure 2.

First, the query matrix of traffic flow is obtained through a linear layer:

(2) $$Q^{f}=W_{q}^{f}X^{f}$$

where $X^{f}$ is the historical input features of traffic flow, $W_{q}^{f}$ is the linear layer weight for obtaining the traffic flow query matrix, and $Q^{f}$ is the traffic flow query matrix.

Then two linear layers are used to obtain the key matrices and value matrices of traffic speed and traffic occupancy, respectively:

(3) $$\left\{\begin{array}{c} K^{s}=W_{k}^{s}X^{s}\\ V^{s}=W_{v}^{s}X^{s} \end{array}\right.$$

(4) $$\left\{\begin{array}{c} K^{o}=W_{k}^{o}X^{o}\\ V^{o}=W_{v}^{o}X^{o} \end{array}\right.$$

where $X^{o}$ and $X^{s}$ are the historical input features of traffic occupancy and traffic speed, respectively, $W_{k}^{s}$ and $W_{k}^{o}$ are the linear layer weights for obtaining the traffic speed and occupancy key matrices, $W_{v}^{s}$ and $W_{v}^{o}$ are the linear layer weights for obtaining the traffic speed and occupancy value matrices, $K^{s}$ and $K^{o}$ are the traffic speed and traffic occupancy key matrices, and $K^{s}$ and $K^{o}$ are the traffic speed and occupancy value matrices.

Then, the prompt values of traffic speed and occupancy are obtained by two cross-attention methods, respectively:

(5) $$\left\{\begin{array}{c} \alpha^{s}=Softmax\left(Q^{f}{\left(K^{s}\right)}^{T}\right)V^{s}\\ \alpha^{o}=Softmax\left(Q^{f}{\left(K^{o}\right)}^{T}\right)V^{o} \end{array}\right.$$

where $\alpha^{s}$ and $\alpha^{o}$ are prompt values of the traffic speed and traffic occupancy.

Finally, the two prompt values are added to the query value of the traffic flow to obtain the feature value with the prompt engineer:

(6) $$H=\alpha^{s}+\alpha^{o}+Q^{f}$$

### 3.2. Feature Extraction

In the feature extraction part, the dual-stream cross structure takes advantage of extracting the spatial–temporal correlation. In one stream branching line, the adaptive graph transformer (AGFormer) takes advantage of extracting the spatial correlation, then the GPT2 network is fine-tuned to extract the temporal correlation, while the other stream branching is reversed.

**Figure 3:**
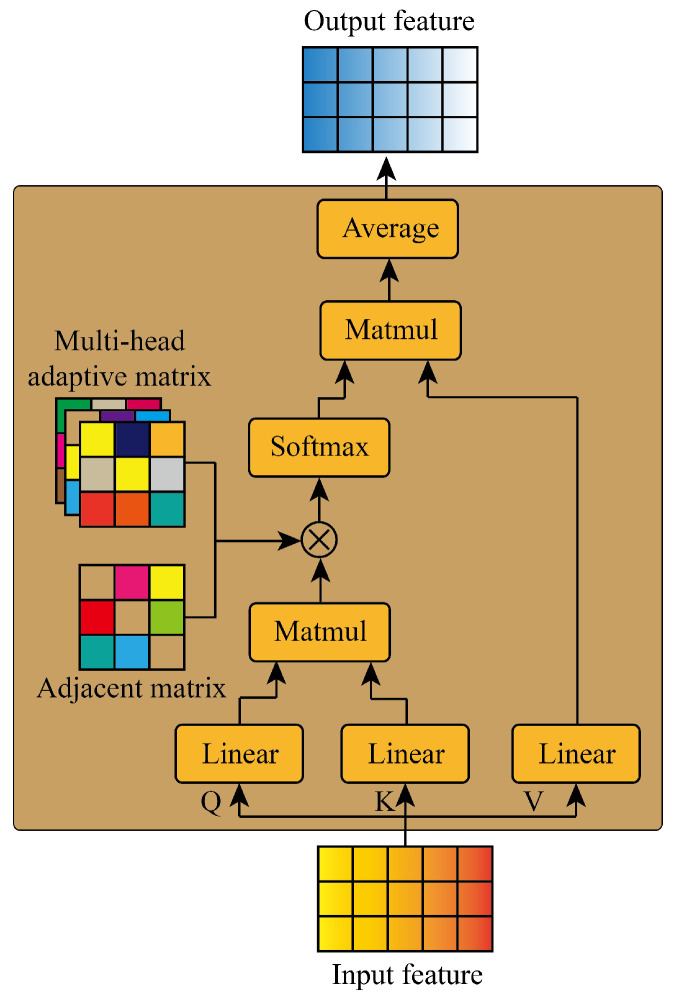
The structure of AGFormer.

AGFormer is a network based on the graph self-attention structure as shown in Figure 3.

The query, key, and value matrices are first obtained through three linear layers:

(7) $$\left\{\begin{array}{c} K^{l}=W_{k}^{l}H^{l}\\ Q^{l}=W_{q}^{l}H^{l}\\ V^{l}=W_{v}^{l}H^{l} \end{array}\right.$$

Then, we obtain the attention coefficient with the adaptive graph structure by obtaining the dot product and the element-wise product:

(8) $$\alpha_{i}^{l}=Sotfmax\left(\left(Q^{l}{\left(K^{l}\right)}^{T}\right)⊗A⊗M_{i}^{l}\right)$$

where *A* is the adjacent matrix, $M_{i}^{l}$ is the *i*th head of the adaptive matrix, and $\alpha_{i}^{l}$ is the *i*th head of the attention coefficient.

Finally, the output features are obtained through averaging:

(9) $$H^{l+1}=\frac{1}{M}\sum_{i=1}^{M}\alpha_{i}^{l}V^{l}$$

where $H^{l+1}$ is the *l* output of the graph self-attention structure, and *M* is the number of the head.

GPT-2 is OpenAI’s second generation generative pre-trained model, introduced in 2019, which consists of multiple layers of transformer encoders, each consisting of multi-head self-attention mechanisms and feedforward neural networks [38]. This architecture allows the model to compute multiple attention weights in parallel to better capture dependencies between different locations and different semantics. By stacking multiple encoder layers, the GPT-2 model is capable of deep feature extraction and representation learning of the input sequence [39].

**Figure 4:**
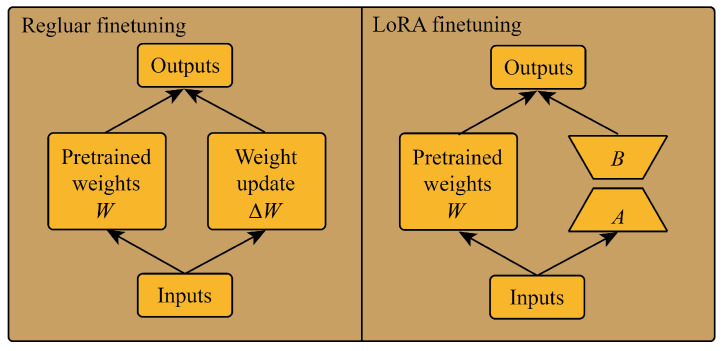
Regular fine-tuning and LoRA fine-tuning.

In order to enable the GPT2 model to mine the temporal correlation of historical traffic information, this paper uses a Low-Rank Adaptation (LoRA) [40] method to fine-tune. The implementation principle of LoRA is to freeze the pre-trained model weights and inject the trainable rank decomposition matrix into each weight in the transformer layer as shown in Figure 4.

Assuming that the update of the weights also has a lower “intrinsic rank” during the adaptation process, for a pre-trained weight matrix $W\in R^{d\times k}$, we represent its updates through low rank decomposition:

(10) W+∇W=W+BA

where $B\in R^{d\times r}$, $A\in R^{r\times k}$, and r≤min(d,k). During the training process, 0 is frozen and does not receive gradient updates, while *A* and *B* contain trainable parameters.

### 3.3. Regression and Model Training

After obtaining the deeply hidden features $H_{F}$ through the dual-stream cross structure, the final prediction values are obtained through the regression module composed of a linear layer. The specific calculation process is as follows:

(11) $$\hat{y}=H_{F}W_{F}$$

where $\hat{y}$ is the final prediction value. $W_{F}$ is the weights of the linear layer.

In order to train the optimal parameters of the above model, the optimizer used for iteration is the Adam algorithm, and the loss function uses the root mean square error, which is calculated as follows:

(12) $$MSE=\frac{1}{NT_{tr}}\sum_{i=1}^{N}\sum_{j=1}^{T_{tr}}{\left({\hat{y}}_{i,j}-y_{i,j}\right)}^{2}$$

where $T_{tr}$ indicates the time size of each training batch, ${\hat{y}}_{i,j}$ indicates the prediction value, and $y_{i,j}$ indicates the true value.

## 4. Experimental Analysis

The entire simulation experiment was completed on a computer with NVIDIA GeForce RTX 3090 (Nvidia, Santa Clara, CA, USA), and the model was built using the Pytorch 2.0.1 open source framework.

Two public data sets in PeMS are used for simulation in this paper [20]. PeMSD4—this data set contains 307 sensor network data for 59 days in the San Francisco Bay Area. PeMSD8—this data set contains 170 sensor network data in the SAN Bernardino area, which were collected over a period of 61 days. In this paper, the data of the last 12 days of the above two data sets are used as the test set, and all the remaining data are used as the training set.

### 4.1. Experimental Parameter Settings

Through several training test experiments, our model parameters with the best performance were selected, setting them as follows: (1) The length of input matrix is 60 min, and the length of the prediction horizon is 60 min. Since the interval between adjacent temporal points is 5 min, $\tau_{1}=\tau_{2}=12$. (2) In the AGFormer module, the number of the head M=3, and the number of the layer is 2. In the GPT2+LoRA module, the size of the rank r=2, and the number of the layer is 6. (3) The batch size is 32, and the learning rate is 1 × $10^{-4}$.

In order to prove the superiority of the model we designed, we use the advanced seven baseline model based on the depth study to compare, which respectively are the LSTM, STGCN [19], ASTGCN [20], STSGCN [23], STFGNN [33], STTNs [25], and STGNCDE [31]. In addition, the LSTM model adopts a five-layer structure. The parameter structure of the other baseline models is set as described in the references.

### 4.2. Comparison of Prediction Performance

First, the prediction accuracy of each prediction model is compared. The mean absolute error (MAE), root mean square error (RMSE), and mean absolute percentage error (MAPE) are used as evaluation metrics:

(13) $$\begin{array}{c} MAE=\frac{1}{NT_{te}}\sum_{i=1}^{N}\sum_{j=1}^{T_{te}}\left|{\hat{y}}_{i,j}-y_{i,j}\right| \end{array}$$

(14) $$\begin{array}{c} RMSE=\sqrt{\frac{1}{NT_{te}}\sum_{i=1}^{N}\sum_{j=1}^{T_{te}}{\left({\hat{y}}_{i,j}-y_{i,j}\right)}^{2}} \end{array}$$

(15) $$\begin{array}{c} MAPE=\frac{100\%}{NT_{te}}\sum_{i=1}^{N}\sum_{j=1}^{T_{te}}\left|\frac{{\hat{y}}_{i,j}-y_{i,j}}{{\hat{y}}_{i,j}}\right| \end{array}$$

where $T_{te}$ is the number of channels in the time dimension of the test set, and ${\hat{y}}_{i,j}$ and $y_{i,j}$ are the predicted and true values. In general, the lower the MAE, RMSE and MAPE, the higher the accuracy of the model.

Table 1 shows MAE, RMSE and MAPE using different prediction models on the two data sets. It can be seen that LSTM has the largest error because it can only mine the time dependence of historical information, and cannot mine the space dependence. STSGCN and STFGNN can simultaneously mine the time and space dependence of historical information, so STGCN and ASTGCN have smaller errors. STTNs and STGNCDE both model the space–time dependence through a new paradigm, so the error is smaller. Our model has the lowest MAE, RMSE and MAPE, indicating that the prediction accuracy of this method is better than other baseline models.

To verify the accuracy of our model over time and space as a whole, the Pearson coefficient and Spearman coefficient are selected as the evaluation indexes of all prediction models. The Pearson coefficient measures the strength and direction of the linear relationship between two variables. Its value range is between −1 and 1: when it approaches 1, it indicates a strong positive correlation between variables; when it approaches −1, it indicates a strong negative correlation between variables; and when it approaches 0, it indicates that there is no linear correlation between variables. The specific calculation formula is:

(16) $$\begin{array}{c} APCT=\frac{1}{T_{te}}\sum_{t=1}^{T_{te}}\frac{E\left[y_{t,:}-\mu_{y_{t,:}}\right)\left({\hat{y}}_{t,:}-\mu_{{\hat{y}}_{t,:}}\right)]}{\sigma_{y_{t,:}\sigma_{{\hat{y}}_{t,:}}}} \end{array}$$

(17) $$\begin{array}{c} APCS=\frac{1}{N}\sum_{n=1}^{N}\frac{E\left[y_{:,n}-\mu_{y_{:,n}}\right)\left({\hat{y}}_{:,n}-\mu_{{\hat{y}}_{:,n}}\right)]}{\sigma_{y_{:,n}\sigma_{{\hat{y}}_{:,n}}}} \end{array}$$

The Spearman coefficient is a non-parametric statistical indicator used to measure the monotonic relationship between two variables. The Spearman coefficient ranges from −1 to 1, similar to the Pearson correlation coefficient. When it approaches 1, it indicates a strong positive correlation between variables; when it approaches −1, it indicates a strong negative correlation between variables; and when it approaches 0, it indicates that there is no monotonic relationship between variables. The specific calculation formula is:

(18) $$\begin{array}{c} ASCT=\frac{1}{T_{te}}\sum_{t=1}^{T_{te}}\left(1-\frac{6\sum_{i=1}^{n}dt_{i}}{nt(nt^{2}-1)}\right) \end{array}$$

(19) $$\begin{array}{c} ASCS=\frac{1}{N}\sum_{n=1}^{N}\left(1-\frac{6\sum_{i=1}^{n}ds_{i}}{ns(ns^{2}-1)}\right) \end{array}$$

Table 2 shows the results of the Pearson correlation coefficient and Spearman correlation coefficient of the eight forecasting models. It can be seen that the two correlation coefficients of our model are closer to 1 than those of the other forecasting models, which means that in terms of time as a whole, the prediction accuracy of the period is the best.

**Figure 5:**
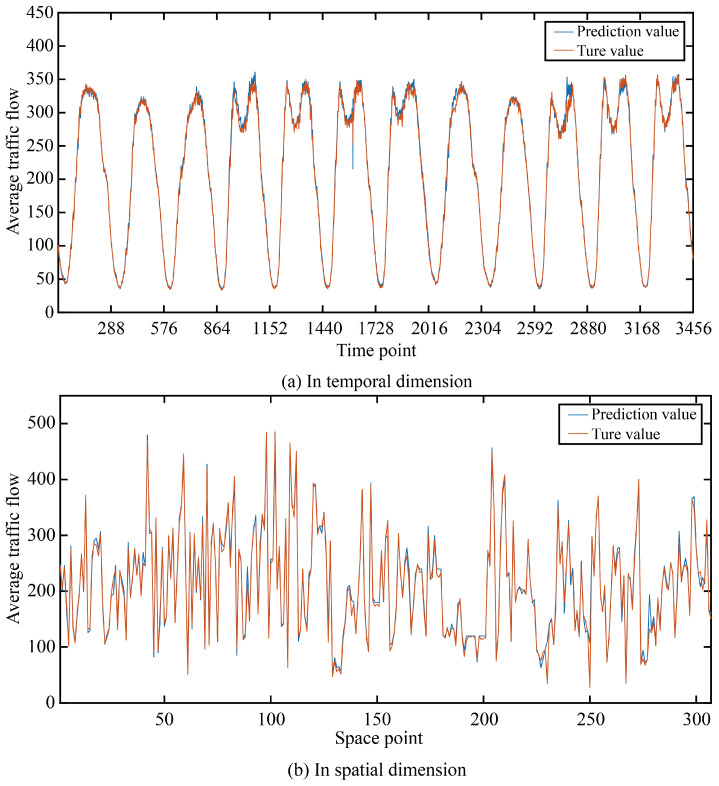
The true value and prediction value on PeMSD4.

**Figure 6:**
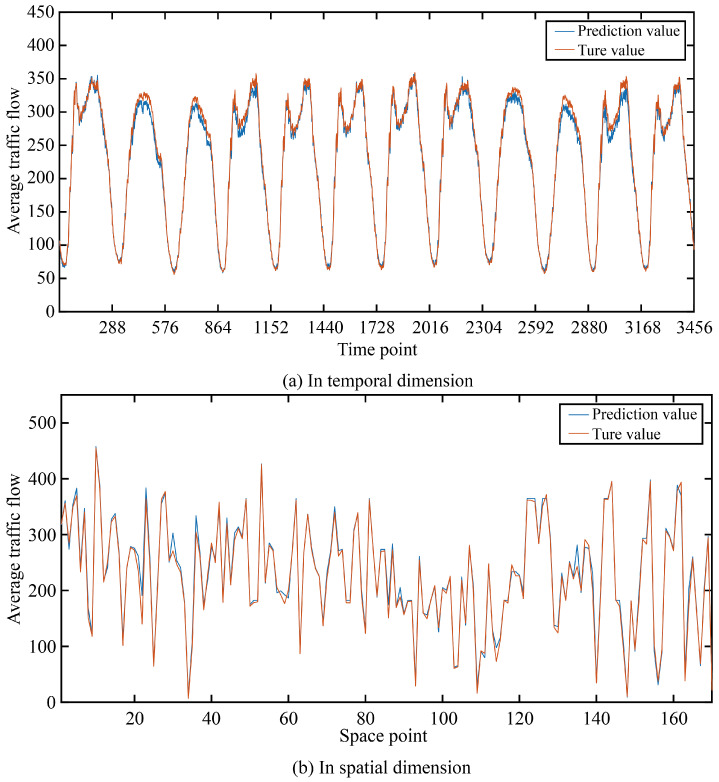
The true value and prediction value on PeMSD8.

In order to more intuitively show the prediction accuracy of our model in the time dimension and space dimension, we visualized the real and predicted values on the two data sets as shown in Figure 5 and Figure 6.

It can be seen that in the two data sets, the real value and the predicted value are very similar in both the time dimension and space dimension, and it can be seen that the accuracy at time points or space points with large average traffic flow is smaller than that at time points or space points with small average traffic flow.

### 4.3. Ablation Experiment

There are two key modules in our model that affect the predictive performance of the model, namely, the prompt engineering module and dual-stream structure.

Firstly, ablation experiments are conducted to verify the impact of the tip engineering module on the performance of the prediction model. Three comparison models are designed. The first ablation model is based on the original model to remove all the suggestive features. The second ablation model is based on the original model to remove the suggestive features provided by the traffic speed. The third ablation model is based on the original model to remove the prompt features provided by the traffic occupancy.

**Figure 7:**
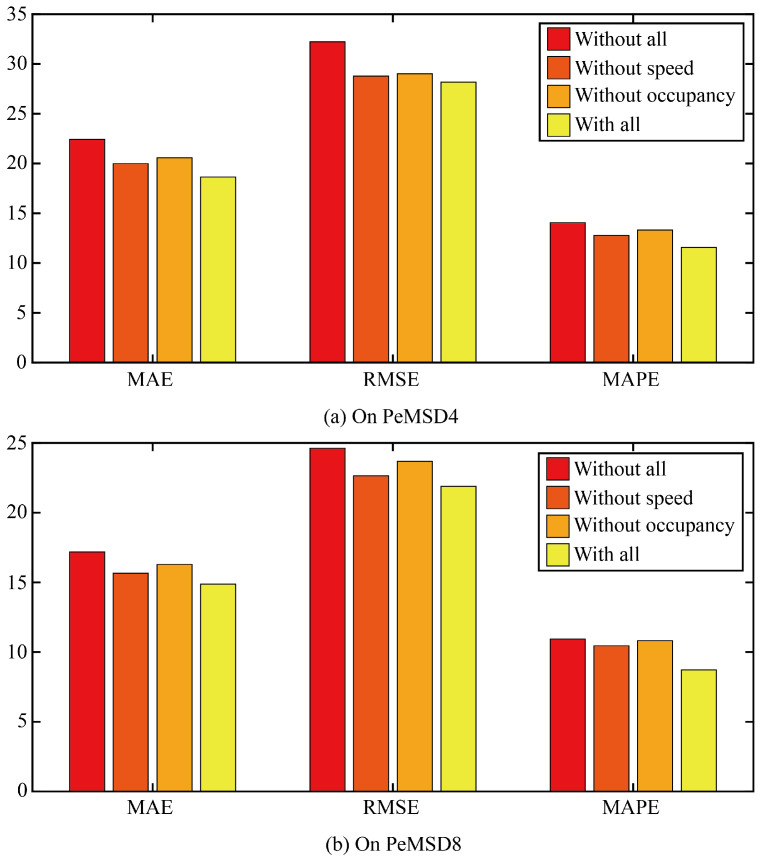
The three error results of the three ablation models and the original model.

The three error results of the three ablation models and the original model are shown in Figure 7. It can be seen that any ablation model can increase the error of the model, indicating that the prompt features of traffic speed and traffic occupancy can increase the prediction accuracy of the model, and the error of removing the traffic speed model is smaller than that of removing the traffic occupancy model. The effect of traffic occupancy on traffic flow is greater than that of traffic speed on traffic flow.

Then, this paper verifies the role of the dual-stream structure through ablation experiments. Two comparison models are designed. In the first ablation model, only the single-stream structure on the left of the original model was retained, that is, the spatial correlation was extracted by AGFomer and then the temporal correlation was extracted by GPT2. The second ablation model only retained the single-stream structure on the right of the original model, that is, GPT2 was first used to extract the temporal correlation, and then AGFomer was used to extract the spatial correlation.

**Figure 8:**
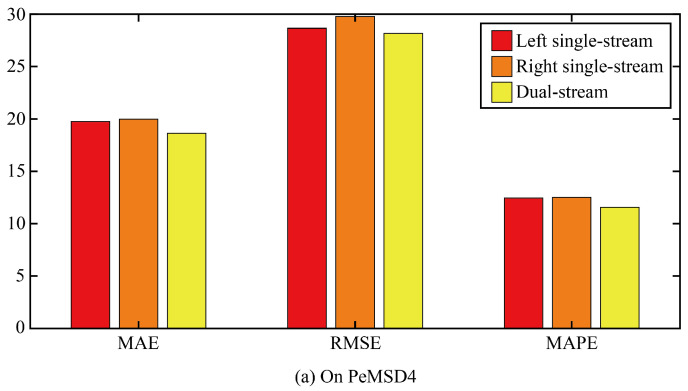

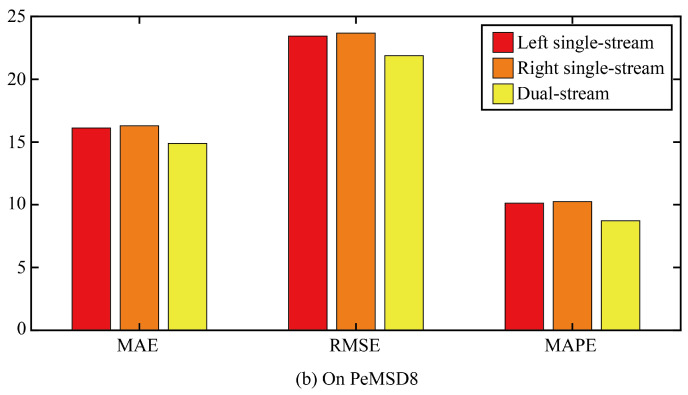
The true value and prediction value on different space points.

The three error results of the three ablation models and the original model are shown in Figure 8. It can be seen that any ablation model can increase the error of the model, which indicates that the dual-stream structure is superior to the single-steam structure in improving the accuracy of the prediction model, and the error similarity of the two ablation models is relatively high, which indicates that the extraction order of the temporal correlation and spatial correlation has little influence on the prediction accuracy of the model.

### 4.4. Real Case Analysis

**Figure 9:**
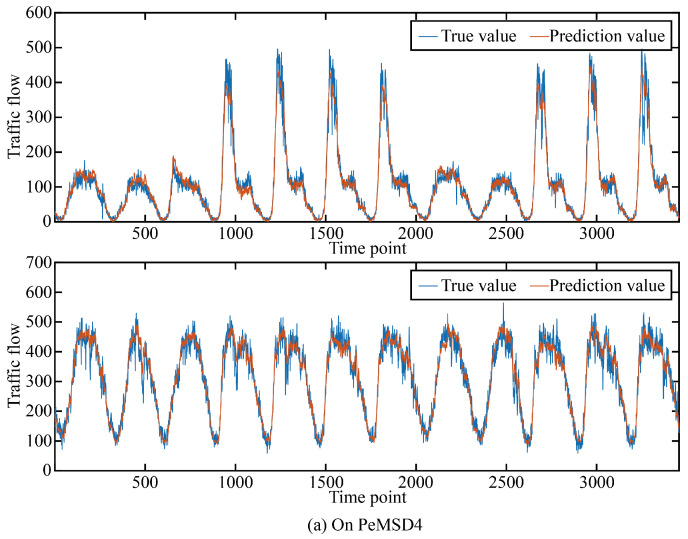

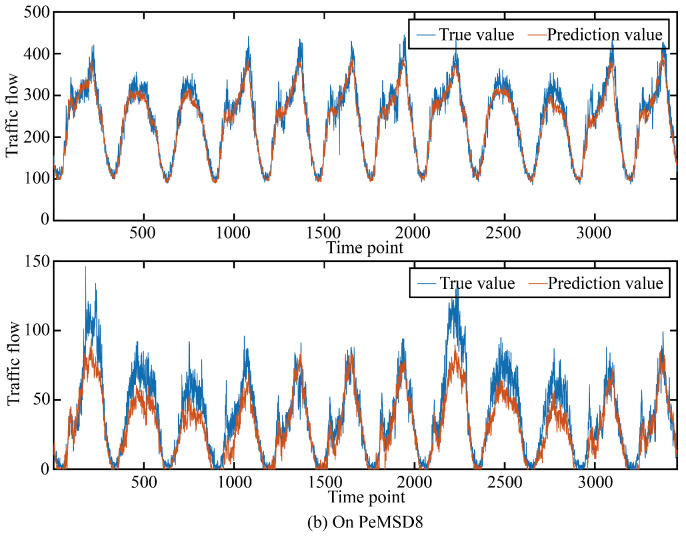
The visualization of real cases.

In this section, in order to verify the validity of our model more directly, we analyze the error of the real value and the calculated value from the aspect of the case study. We randomly select two spatial points from two data sets and visualize their predicted and true values as shown in Figure 9. We randomly select two space points from two data sets and visualize their predicted value and true value as shown in Figure 9. It can be seen that the predicted value of our model still maintains a small error under different conditions, but the error will be slightly larger when the fluctuation is large.

## 5. Conclusions

In this paper, a dual-stream cross AGFormer-GPT network with prompt engineering is proposed for traffic flow prediction, which integrates traffic occupancy and traffic speed as prompt features into traffic flow through the form of cross-attention, and uses the dual-stream cross structure to uniquely mine spatial and temporal correlation information, effectively capturing the complex dynamics of traffic patterns. In terms of three types of errors, our model is decreased by 0.57, 2.94, and 1.21% compared to the most accurate comparison model on the PeMS04 data set, and by 0.58, 2.93, and 1.20% on the PeMS08 data set. In addition, we verify the importance of key modules through ablation experiments.

## Figures and Tables

**Table 1:** Three error indicators of different prediction models on two data sets.

	PeMSD4			PeMSD8		
	**MAE**	**RMSE**	**MAPE**	**MAE**	**RMSE**	**MAPE**
LSTM	29.32	45.72	18.59%	23.10	36.89	13.15%
STGCN	27.02	38.16	14.13%	20.70	30.32	10.64%
ASTGCN	21.80	32.82	12.43%	16.63	25.27	9.40%
STSGCN	21.24	33.70	14.02%	16.90	26.31	10.80%
STFGNN	19.83	31.87	13.02%	16.64	26.21	10.55%
STTNs	19.48	31.91	13.63%	15.48	24.97	10.13%
STGNCDE	19.21	31.09	12.77%	15.46	24.81	9.92%
Ours	18.64	28.15	11.56%	14.88	21.88	8.72%

**Table 2:** Correlation coefficient of different prediction models on two data sets.

	PeMSD4				PeMSD8			
	**APCT**	**APCS**	**ASCT**	**ASCS**	**APCT**	**APCS**	**ASCT**	**ASCS**
LSTM	0.9275	0.9277	0.9062	0.9195	0.9441	0.9507	0.9268	0.9459
STGCN	0.9308	0.9358	0.9097	0.9289	0.9487	0.9604	0.9316	0.9564
ASTGCN	0.9476	0.9638	0.9299	0.9594	0.9628	0.9740	0.9510	0.9693
STSGCN	0.9490	0.9645	0.9325	0.9602	0.9615	0.9732	0.9494	0.9685
STFGNN	0.9557	0.9647	0.9403	0.9598	0.9641	0.9751	0.9751	0.9706
STTNs	0.9571	0.9629	0.9427	0.9590	0.9683	0.9766	0.9581	0.9722
STGNCDE	0.9582	0.9710	0.9439	0.9666	0.9678	0.9774	0.9576	0.9732
Ours	0.9604	0.9719	0.9472	0.9675	0.9687	0.9781	0.9585	0.9739

## Data Availability

Data set download URL: https://gitcode.com/wanhuaiyu/ASTGCN/tree/master/data.
